# A risk scoring system to predict progression to severe pneumonia in patients with Covid-19

**DOI:** 10.1038/s41598-022-07610-9

**Published:** 2022-03-30

**Authors:** Ji Yeon Lee, Byung-Ho Nam, Mhinjine Kim, Jongmin Hwang, Jin Young Kim, Miri Hyun, Hyun Ah Kim, Chi-Heum Cho

**Affiliations:** 1 Department of Infectious Diseases, Keimyung University Dongsan Hospital, Keimyung University School of Medicine, Daegu, Republic of Korea; 2 Covid-19 Task Force Team of Keimyung University Daegu Dongsan Hospital, Daegu, Republic of Korea; 3 HERINGS, Institute of Advanced Clinical and Biomedical Research, Seoul, Republic of Korea; 4 Division of Health Policy and Administration, School of Public Health, University of Illinois at Chicago, Chicago, USA; 5 Department of Cardiology, Keimyung University Dongsan Hospital, Keimyung University School of Medicine, Daegu, Republic of Korea; 6 Department of Radiology, Keimyung University Dongsan Hospital, Keimyung University School of Medicine, Daegu, Republic of Korea; 7 Department of Obstetrics and Gynecology, Keimyung University Dongsan Hospital, Keimyung University School of Medicine, 1035, Dalgubeol-daero, Dalseo-gu, Daegu, 42601 Republic of Korea

**Keywords:** Diseases, Infectious diseases, Viral infection

## Abstract

Rapid outbreak of coronavirus disease 2019 (Covid-19) raised major concern regarding medical resource constraints. We constructed and validated a scoring system for early prediction of progression to severe pneumonia in patients with Covid-19. A total of 561 patients from a Covid-19 designated hospital in Daegu, South Korea were randomly divided into two cohorts: development cohort (N = 421) and validation cohort (N = 140). We used multivariate logistic regression to identify four independent risk predictors for progression to severe pneumonia and constructed a risk scoring system by giving each factor a number of scores corresponding to its regression coefficient. We calculated risk scores for each patient and defined two groups: low risk (0 to 8 points) and high risk (9 to 20 points). In the development cohort, the sensitivity and specificity were 83.8% and 78.9%. In the validation cohort, the sensitivity and specificity were 70.8% and 79.3%, respectively. The C-statistics was 0.884 (95% CI 0.833–0.934) in the development cohort and 0.828 (95% CI 0.733–0.923) in the validation cohort. This risk scoring system is useful to identify high-risk group for progression to severe pneumonia in Covid-19 patients and can prevent unnecessary overuse of medical care in limited-resource settings.

## Introduction

After the Coronavirus disease 2019 (Covid-19) first occurred in China on December 2019, it has become pandemic in March^1^. Due to the surge in Covid-19 patients, lack of medical resources (e.g., negative pressure rooms, personal protective equipment and medical personnel) has become a major problem. During the height of the outbreak in early March, the Ministry of Health and Korea Centers for Disease Control and Prevention established a policy guideline of assigning patients with Covid-19 to different treatment centers based on the level of severity and risk in order to maximize the usage of medical resources. Patients with mild symptoms were assigned to community treatment centers to recover whereas high-risk patients with old age or moderate to severe cases were hospitalized. However, there has been an increasing number of patients classified as mild cases at admission who have developed severe pneumonia during hospitalization.

It is important to provide timely critical care to Covid-19 patients with serious symptoms to reduce number of deaths and burden on overall health system^2^. Therefore, it is key to identify patients who are at higher risk of developing severe pneumonia in early stages of disease. Previous studies show that established and potential risk factors associated with Covid-19 complications are older age (e.g., > 65 years), cardiovascular disease, chronic lung disease, diabetes mellitus, obesity, immunocompromise, end-stage renal disease, and liver disease^3–7^. The purpose of this study is to present a novel scoring system that could be used by clinicians to predict progression to severe pneumonia in Covid-19 patients in earlier stages.

## Methods

### Study population

Keimyung University Daegu Dongsan Hospital (KDDH) were designated as Covid-19 regional hospital on February 21, 2020 during a large outbreak of Covid-19 in Daegu, South Korea. We enrolled all patients (≥ 18 years old) with confirmed Covid-19 admitted to this hospital from February 21, 2020 to March 31, 2020. Patients presenting with severe Covid-19 pneumonia at admission were excluded. A Covid-19 patient was defined as an individual with laboratory confirmation of Covid-19 which required positive results of SARS-CoV-2 RNA, irrespective of clinical signs and symptoms. Severe Covid-19 pneumonia was defined as at least one of the followings: (1) resting oxygen saturation ≤ 93% in room air, or (2) PaO2/FiO2 ≤ 300 mmHg or requirement of mechanical ventilation^8^. Patients were classified into two groups: Progression (progression to severe pneumonia during hospitalization) and Stable (not requiring oxygen treatment during hospitalization). All patients with stable Covid-19 infection during hospitalization were followed for more than 4 weeks after admission. The study was conducted in accordance with the Declaration of Helsinki. Informed written consent was obtained from all participants. This study was approved by the Institutional Review Board at the Keimyung University Dongsan Hospital (IRB No. 2020-03-027).

### Data collection

The presenting history, comorbidity status, epidemiologic history, and vital signs were collected. Comorbidities were defined as having at least one of the followings: hypertension, diabetes mellitus, hyperlipidemia, cardiovascular disease, chronic pulmonary disease, chronic liver disease, chronic kidney disease, neurologic disease, autoimmune disease, malignancy and HIV infections. The laboratory parameters, including complete blood count, liver and renal function, C-reactive protein (CRP), lactate dehydrogenase (LDH), creatinine phosphokinase (CPK) were examined at admission. The oxygen saturation was measured by pulse oxygen saturation on room air at rest state. All patients were examined by chest X-ray. Clinical outcome (progression of illness) were monitored up to April 26, 2020. Data were collected retrospectively by electronic medical chart review.

### Statistical analysis

Potential risk factors for progression to severe pneumonia were evaluated. The Wilcoxon rank sum test was used for continuous variables and the Chi-square test was used for categorical variables to examine the baseline difference between stable and progressed patient group. Most continuous variables that showed significant difference at the baseline were dichotomized. The cut-off values for each continuous variable were determined such that it showed the best discriminatory ability based on the Youden index (sensitivity + specificity − 1). Age group and LDH group were categorized based on the risk probability trend of specific sections within age (by 5 years) and LDH (50 U/L). Age-adjusted logistic regression model was used to identify risk factors. Multivariate logistic regression model with stepwise selection process was used to develop a risk prediction model. *P* value of 0.05 was used for variable selection process. The model performance was evaluated with respect to its discrimination and calibration ability. Discrimination was quantified using the C-statistics and Hosmer–Lemeshow (H–L) χ^2^ statistic was calculated for calibration. The Receiver Operating Characteristics (ROC) Curve for the C-statistics was generated and the square distance between observed prevalence and mean predicted probabilities for each quintile of predicted risk was assessed for calibration. The model performance was also evaluated on the separate validation cohort. Statistical analyses were performed using SAS version 9.4 (SAS institute, Cary, NC), and R package version 3.6.2.

## Results

### Clinical characteristics of patients

**Figure 1:**
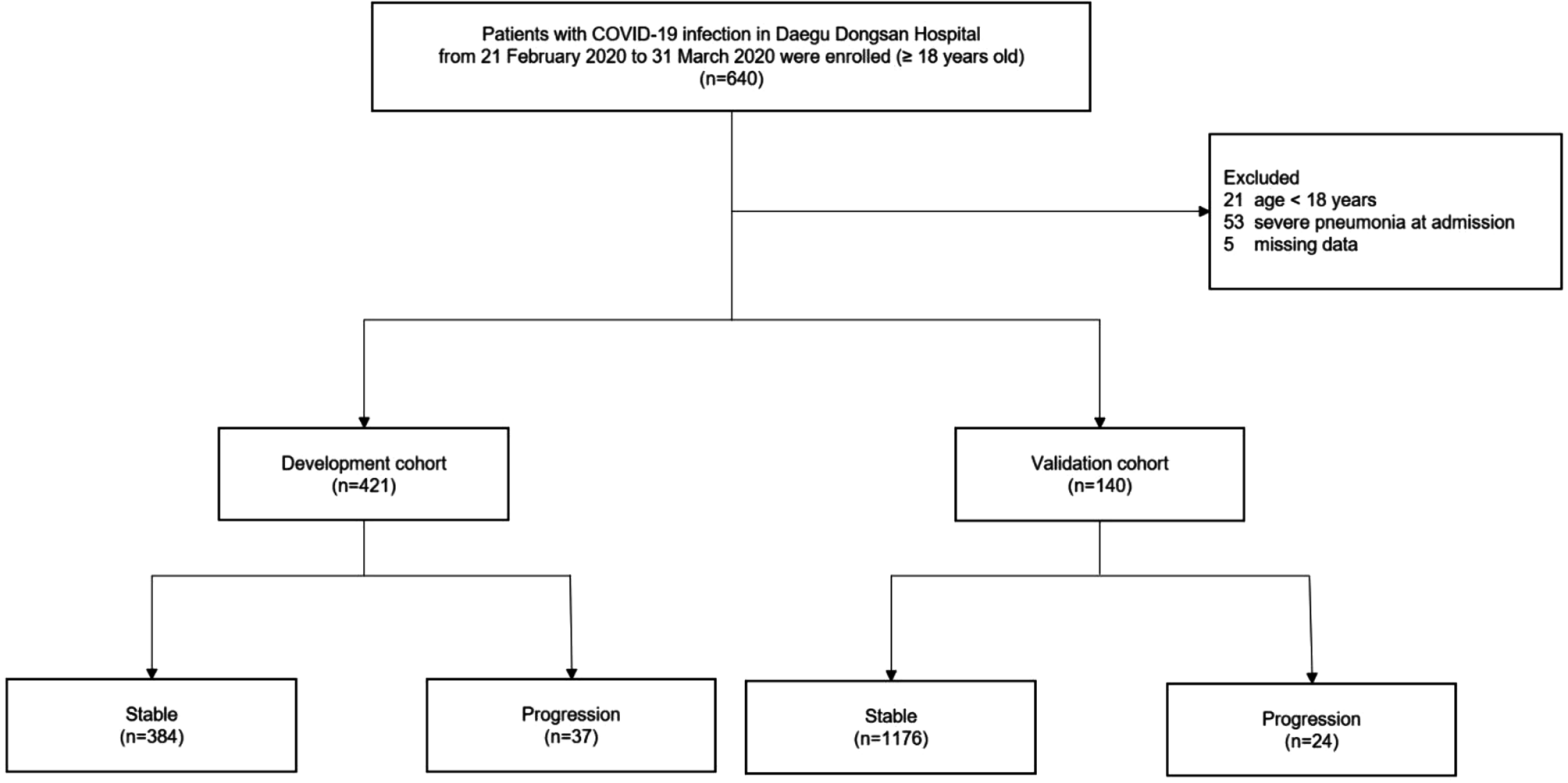
Selection of study patients.

The selection of the study population is illustrated in Fig. 1. A total 640 patients were hospitalized from February 21 through March 31, 2020, the follow-up period ended in April 26, 2020. 21 cases were excluded for the age under 18 years old, 53 patients were excluded for severe pneumonia at admission, and 5 patients were excluded for lack of laboratory examination. Out of 561 patients, 421 were randomly placed in the development cohort and 140 were assigned to the validation cohort, respectively. All patients with stable Covid-19 during hospitalization were followed for more than 4 weeks after admission. Demographics and clinical characteristics of patients who remained stable and who had progressed to severe pneumonia in Covid-19 infection are summarized in Supplementary Table 1 for the development cohort and Supplementary Table 2 for the validation cohort. In the development cohort, 37 (8.8%) of 421 patients had progressed to severe pneumonia. In the validation cohort, 24 patients (17.1%) developed severe Covid-19 pneumonia.

### Independent risk factors associated with progression to severe pneumonia

A risk prediction model was developed in the development cohort. In the univariate analysis, age, male gender, comorbidities, initial chest X-ray abnormality, absolute neutrophil count (ANC), absolute lymphocyte count (ALC), platelet count, blood urea nitrogen (BUN), estimated glomerular filtration rate (eGFR), aspartate aminotransferase (AST), albumin, CRP, CPK and LDH were significantly associated with progression to severe pneumonia.

The age-adjusted univariate analysis revealed male gender, comorbidities, initial chest X-ray abnormality, ALC, platelet, BUN, AST, albumin, CRP, CPK and LDH as significantly associated factors. Age, hemoglobin, CRP and LDH were included in the final model by stepwise selection process in the multivariate logistic regression model (Table 1). In the development cohort, the proportions of progression to severe pneumonia in each category were seen as follows: age [< 50 years (0.7%), 50–59 years (6.0%), 60–69 years (10.3%), 70–79 years (17.6%), ≥ 80 years (37.5%)], hemoglobin [< 13 g/L (7.5%), ≥ 13 g/L (11.3%)], CRP [< 1.4 mg/dL (4.0%), ≥ 1.4 mg/dL (26.1%)], LDH [< 500 U/L (3.9%), 500–700 U/L (15.1%), ≥ 700 U/L (45.8%)]. In the validation cohort, the respective proportions were : age [< 50 years (6.5%), 50–59 years (16.7%), 60–69 years (26.7%), 70–79 years (13.6%), ≥ 80 years (66.7%)], hemoglobin [< 13 g/L (13.7%), ≥ 13 g/L (24.4%)], CRP [< 1.4 mg/dL (8.3%), ≥ 1.4 mg/dL (46.9%)], LDH [< 500 U/L (10.6%), 500–700 U/L (18.2%), ≥ 700 U/L (61.5%)] (Supplementary Fig. 1).

**Table 1:** Univariate and multiple logistic regression analysis in the development cohort.

Risk factor	Univariate analysis		Age-adjusted univariable analysis		Multiple analysis	
	OR (95% CI)	*p* value	OR (95% CI)	*p* value	OR (95% CI)	*p* value
Age, years						
< 50	1.00 (Ref.)					
50–59	9.00 (1.51–171.42)	0.043			6.63 (0.75–58.96)	0.090
60–69	16.27 (2.98–302.81)	0.009			7.93 (0.91–69.41)	0.061
70–79	30.21 (5.75–556.93)	0.001			24.99 (2.91–214.99)	0.003
≥ 80	84.60 (14.50 – 1616.18)	< 0.001			69.28 (7.45–644.60)	< 0.001
Gender, male						
Female	1.00 (Ref.)		1.00 (Ref.)			
Male	3.08 (1.55–6.15)	0.001	3.20 (1.53–6.77)	0.002		
Comorbidities						
Without	1.00 (Ref.)		1.00 (Ref.)			
With	3.70 (1.80–8.23)	0.001	1.33 (0.58–3.19)	0.002		
Initial CXR						
Normal	1.00 (Ref.)		1.00 (Ref.)			
Lung infiltration	6.99 (3.16–17.68)	< 0.001	4.30 (1.87–11.26)	0.001		
ANC, /μL						
< 3010	1.00 (Ref.)		1.00 (Ref.)			
≥ 3010	2.91 (1.46–6.06)	0.003	1.62 (0.76–3.56)	0.218		
ALC, /μL						
≥ 1000	1.00 (Ref.)		1.00 (Ref.)			
< 1000	4.64 (2.10–9.88)	< 0.001	3.14 (1.34–7.11)	0.007		
Hemoglobin, g/dL						
< 13.3	1.00 (Ref.)		1.00 (Ref.)			
≥ 13.3	1.58 (0.79–3.12)	0.191	2.95 (1.36–6.46)	0.006	3.79 (1.30–7.31)	0.011
Platelet, 10^3^/uL						
≥ 150	1.00 (Ref.)		1.00 (Ref.)			
< 150	2.84 (1.19–6.27)	0.013	3.08 (1.21–7.45)	0.014		
BUN, mg/dL						
< 19	1.00 (Ref.)		1.00 (Ref.)			
≥ 19	4.59 (2.16–9.48)	< 0.001	2.35 (1.04–5.11)	0.035		
eGFR, mL/min/1.73 m^2^						
≥ 60	1.00 (Ref.)		1.00 (Ref.)			
< 60	5.74 (2.06–14.77)	< 0.001	2.30 (0.76–6.43)	0.121		
AST, U/L						
< 30	1.00 (Ref.)		1.00 (Ref.)			
≥ 30	3.88 (1.92–7.80)	< 0.001	4.20 (1.93–9.29)	< 0.001		
ALT, U/L						
< 19	1.00 (Ref.)		1.00 (Ref.)			
≥ 19	1.48 (0.75–3.03)	0.268	1.93 (0.90–4.40)	0.103		
Albumin, g/dL						
≥ 4.2	1.00 (Ref.)		1.00 (Ref.)			
< 4.2	6.14 (2.55–18.25)	< 0.001	3.21 (1.27–9.87)	0.023		
CRP, mg/dL						
< 1.4	1.00 (Ref.)		1.00 (Ref.)			
≥ 1.4	8.58 (4.22–18.17)	< 0.001	6.17 (2.88–13.78)	< 0.001	3.79 (1.52–9.44)	0.004
CPK, U/L						
< 69	1.00 (Ref.)		1.00 (Ref.)			
≥ 69	2.31 (1.17–18.17)	< 0.001	2.15 (1.04–4.50)	0.039		
LDH, U/L						
< 500	1.00 (Ref.)		1.00 (Ref.)			
500–700	4.31 (1.92–9.85)	< 0.001	3.34 (1.41–8.07)	0.006	2.12 (0.82–5.48)	0.119
≥ 700	20.59 (7.68–56.45)	< 0.001	17.33 (5.89–52.93)	< 0.001	7.21 (2.12–24.56)	0.002

ALC, absolute lymphocyte count; ALT, Alanine aminotransferase; ANC, absolute neutrophil count; CI, confidence interval; AST, Aspartate aminotransferase; BUN, blood urea nitrogen; CPK, Creatinine phosphokinase; CRP, C-reactive protein; CXR, chest X-ray; eGFR, estimated glomerular filtration rate; LDH, lactate dehydrogenase; OR, odds ratio.

### Construction and performance of KDDH scoring system

A scoring system was developed such that the points given to each variable correspond to its regression coefficients in the model (Table 2). The maximum points that a patient can get is 20. This scoring system was constructed based on the four factors, and two patient groups were constructed based on risk scores: low risk (0 to 8 points) and high risk (9 to 20 points). We identified patient group with over the score of 9 to have higher possibility of developing severe pneumonia. Sensitivity and specificity in the development cohort were 83.8% and 78.9%, respectively. In the development cohort, 31 (27.7%) of high-risk group and 6 (1.9%) of low-risk group progressed to severe pneumonia.

**Table 2:** The calculator of KDDH score.

Parameter	Points
Age, years	
< 50	0
50–59	4
60–69	5
70–79	7
≥ 80	10
C-reactive protein, mg/dL	
< 1.4	0
≥ 1.4	3
Lactate dehydrogenase, U/L	
< 500	0
500–700	2
≥ 700	4
Hemoglobin, g/dL	
< 13.3	0
≥ 13.3	3

**Figure 2:**
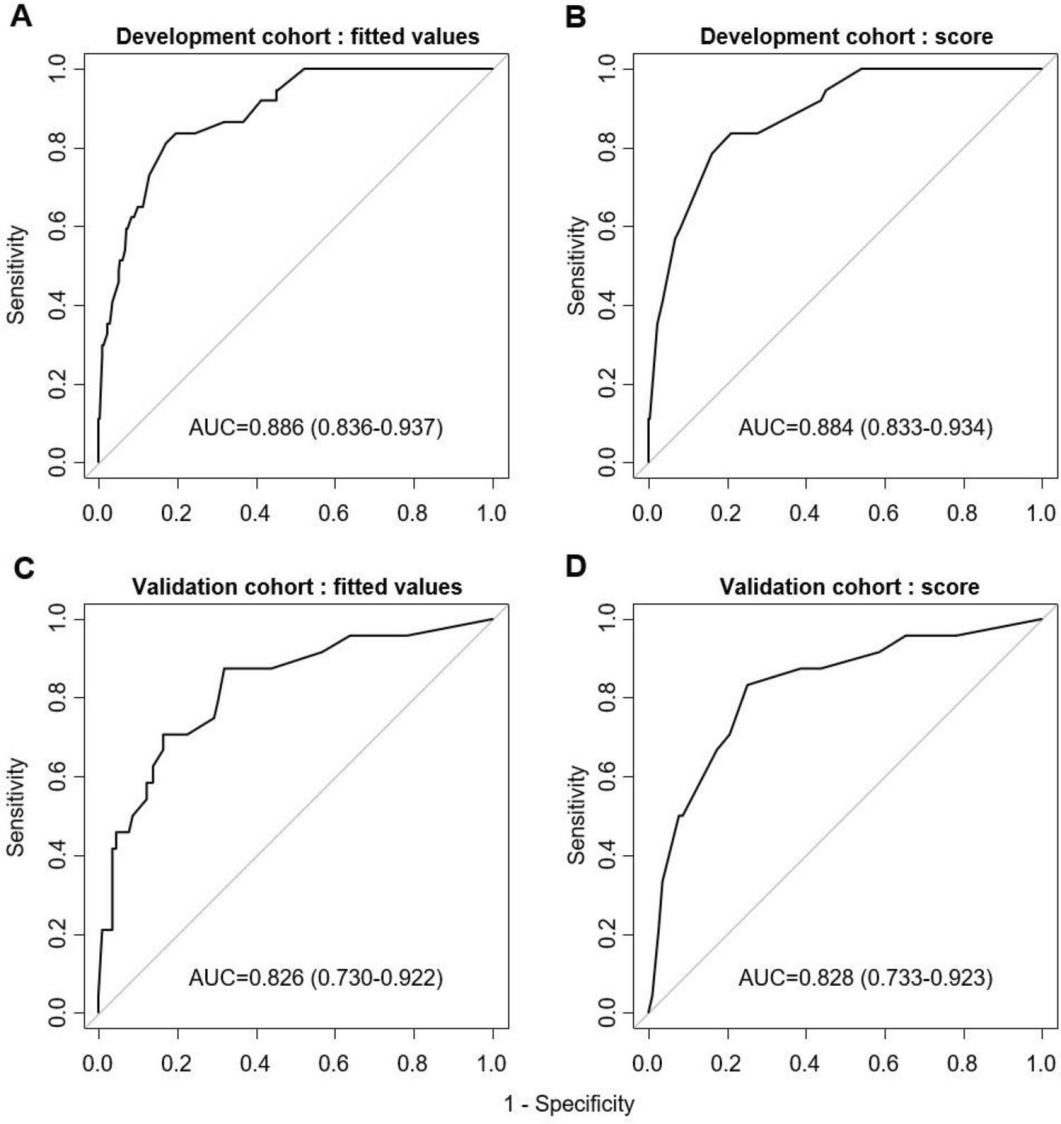
Discrimination ability of the severe pneumonia in prediction model in patients with Covid-19. Receiver operating characteristics (ROC) curves for predictive value in the (**A**) development cohort: fitted value, (**B**) development cohort: KDDH score, (**C**) validation cohort: fitted value, and (**D**) validation cohort: KDDH score.

The discrimination ability was evaluated for both prediction model and score system in terms of C-statistics. The C-statistics for the model was 0.886 (95% CI 0.836–0.937) and 0.884 (95% CI 0.833–0.934) for the score system (Fig. 2).

### Validation of the risk prediction models

**Figure 3:**
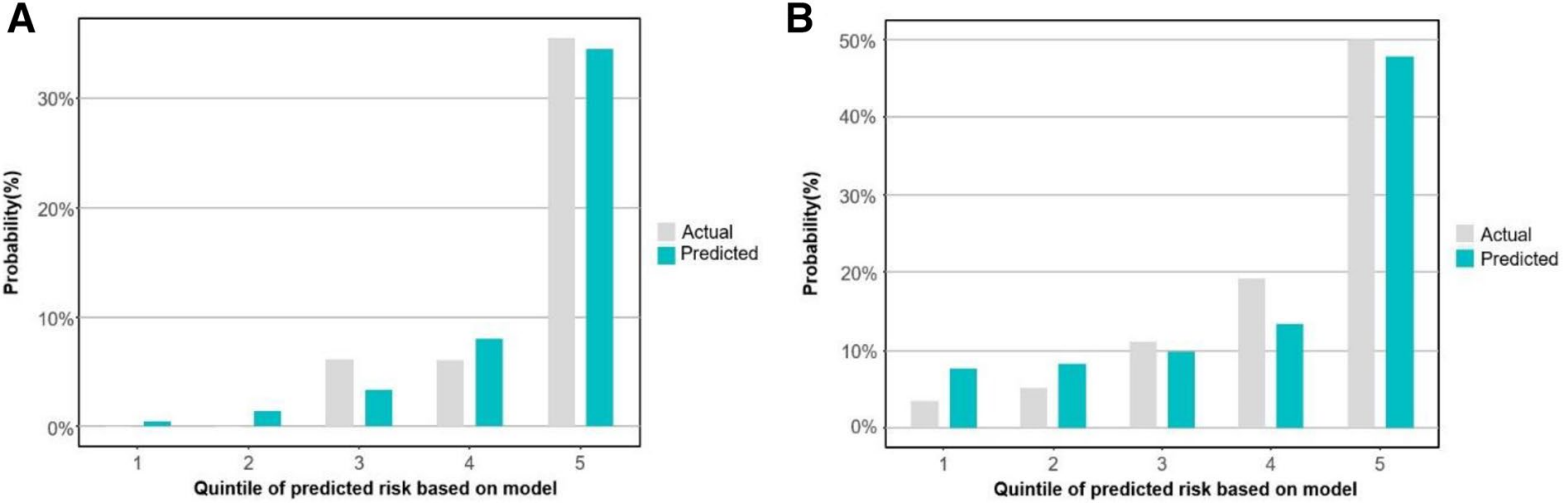
Calibration ability of the severe pneumonia prediction model in patients with Covid-19. The calibration bar plot in the development cohort (**A**) and the validation cohort (**B**).

In the validation cohort, 17 (41.5%) of high-risk group and 7 (7.1%) of low-risk group developed severe pneumonia. Baseline characteristics for the validation cohort were similar to those in the development cohort (Supplementary Table 2). At the cut-off value of 9 points for high-risk group, sensitivity and specificity of score system in the development cohort were 70.8% and 79.3%, respectively (Table 3). The C-statistics were 0.826 (95% CI 0.0.730–0.922) for the fitted value model and 0.828 (95% CI 0.733–0.923) for the scoring system in the validation cohort (Fig. 2). The model calibration on the validation cohort are shown in the Fig. 3. There was no significant difference between predicted progression to severe pneumonia with KDDH score and actual progression (χ^2^ = 2.448, *p* value = 0.485).

**Table 3:** Performance for early prediction of severe Covid-19.

	Development cohort (n = 421)		Validation cohort (n = 140)	
AUC (95% CI)				
Fitted value	0.886 (0.836–0.937)		0.826 (0.730–0.922)	
KDDH Score	0.884 (0.833–0.934)		0.828 (0.733–0.923)	
Cut-off Points 9 (stable/progression)				
High-risk (Points ≥ 9)	31 TP (27.7%)	81 FP (72.3%)	17 TP (41.5%)	24 FP (58.5%)
Low-risk (Points < 9)	6 FN (1.9%)	303 TN (98.1%)	7 FN (7.1%)	92 TN (92.9%)
Sensitivity (%)	83.8%		70.8%	
Specificity (%)	78.9%		79.3%	
Positive predictive value (%)	27.7%		41.5%	
Negative predictive value (%)	98.1%		92.9%	
Positive likelihood ratio	3.972		3.424	
Negative likelihood ratio	0.206		0.368	

FN, false negative; FP, false positive; TN, true negative; TP, true positive.

## Discussion

This study presented a novel scoring system which can be used to predict progression of Covid-19. Identifying Covid-19 patients who are at a higher risk of developing severe symptoms in early stages can inform better medical resource allocation and patient care during massive outbreaks. We found age, CRP, LDH and hemoglobin as independent high-risk factors associated with progression of Covid-19 infection. Some of our findings are consistent with previous studies that identified different risk factors to be associated with poor clinical outcomes in patients with Covid-19^6,7,9,10^. Older age, high Sequential Organ Failure Assessment (SOFA) score, and d-dimer greater than 1 µg/mL at admission were described as potential risk factors for mortality of in-hospital patients with Covid-19^6^. Wang et al.^9^ reported older age, dyspnea, lymphopenia, comorbidities (e.g., cardiovascular disease), and acute respiratory distress syndrome (ARDS) as predictors of fatal outcomes in elderly Covid-19 patients. Age, sex, CRP, LDH, lymphocyte count, and features derived from CT images were most reported predictors of severe Covid-19 progression^10^. Jiang X et al. presented research on progression to ARDS through AI framework, and it mentioned higher hemoglobin as one of the risk factors of later development of ARDS. Moreover, higher hemoglobin levels were associated male gender or even unreported tobacco use^11^.

To our knowledge, KDDH scoring system is one of few scoring systems to predict Covid-19 progression at early stage. Ji et al.^12^ created a CALL score model with age, comorbidities, lymphopenia and LDH to predict Covid-19 progression at early stage. This is a relatively simple scoring system that only require basic tests for laboratory parameters. However, it has limitations of smaller sample size and no validation. There exists difference in definition of progression to severe Covid-19. CALL model defines deteriorated chest radiologic findings in progression, but the present study only defines patients requiring oxygen therapy as progression^12^. We did not include progression of radiologic findings without hypoxemia in the progression group, as deterioration of radiologic finding tends to be reflected later than clinical course. Gong et al.^13^ presented a prognostic nomogram based on seven factors (older age, higher LDH and CRP, direct bilirubin, red blood cell distribution width (RDW), BUN, and lower albumin) to identify patients who are likely to develop severe Covid-19 infection. The nomogram was conducted with multicenter patients and was validated. However, its scoring system shows limited applicability due to score model complexity and greater number of clinical parameters. Liang et al.^14^ developed a clinical risk score to predict risk of developing critical illness in Covid-19 patients, which was not associated with progression to severe pneumonia.

The KDDH scoring system has advantages of high sensitivity and specificity together with strong calibration that ensures no statistical difference between quintile of predicted risk. Moreover, this is uncomplicated and can predict progression to severe pneumonia by using a simple blood test that can be conducted in outpatient clinics.

High negative predictive value (98.1% in development cohort & 92.9% in validation cohort) of the presented scoring system demonstrates efficacy in identifying low risk patients, who can be managed at other treatment facilities with minimal monitoring or through self-quarantine. High-risk group who are likely to require oxygen therapy can be assigned first to hospitals and receive priority care in early stage. With this scoring system, hospitals can prevent unnecessary overuse of medical care in limited resource settings (e.g., during massive outbreaks), and may reduce mortality through effective allocation of medical resources. During the peak of the Covid-19 outbreak in South Korea, several patient deaths occurred in their homes while waiting to be hospitalized after receiving Covid-19 diagnosis due to shortage of beds. By triaging patients using the KDDH scoring system, such mortality cases could be reduced and be prevented in future outbreaks.

This study has several limitations. First, some study showed that severity of COVID-19 infection is related with smoking, obesity, time between symptoms and hospital admission^15,16^. But there is no data collection of these variables in this study. Second, this study has limitation of being a retrospective, single center cohort study conducted in Daegu, South Korea. However, this hospital treated the largest number of patients with Covid-19, thus its patient data is well-representative of the entire country. Moreover, it is one of the few studies conducted outside of China to develop and validate a risk scoring model for Covid-19 patients. A substantial number of patients were enrolled and followed up for more than 4 weeks without any change observed in final patient outcomes, and the scoring system was well-validated.

In our experience, based on the KDDH scoring system, the low-risk patients received care in the mild patient ward where oxygen therapy was unavailable. The high-risk patients were admitted in the main ward where oxygen therapy and close monitoring could be provided. By concentrating medical personnel who can provide critical care at the main ward, we could treat the critically ill patents effectively.

In summary, the presented KDDH scoring system was validated to be a highly informative and successful risk stratification tool to identify Covid-19 patients at higher risk of progression to severe pneumonia. Early adoption of this scoring system can assist optimal usage of limited medical resources in different health facility settings that are undergoing rapid Covid-19 outbreaks.

## Supplementary Information

Supplementary Information.

## Data Availability

The datasets generated and/or analyzed during the present study are available from the corresponding author on reasonable request.
