# Supplementary material for: A risk scoring system to predict progression to severe pneumonia in patients with Covid-19
Source: Sci Rep. 2022 Mar 30;12:5390. doi: 10.1038/s41598-022-07610-9 (PMC8966605; doi:10.1038/s41598-022-07610-9)

**A Risk Scoring System to Predict Progression to Severe Pneumonia in Patients with Covid-19**

Ji Yeon Lee, MD, PhD^1,2^, Byung-Ho Nam, PhD^3^, Mhinjine Kim, MHS^2,4^, Jongmin Hwang, MD, PhD^5^, Jin Young Kim, MD^6^, Miri Hyun, MD, PhD^1,2^, Hyun Ah Kim, MD, PhD^1,2^, Chi-Heum Cho, MD, PhD^2,7*^

^1^Department of Infectious disease, Keimyung University Dongsan Hospital, Keimyung University School of Medicine, Daegu, Republic of Korea

^2^Covid-19 Task Force Team of Keimyung University Daegu Dongsan Hospital, Daegu, Republic of Korea

^3^HERINGS, Institute of Advanced Clinical & Biomedical Research, Seoul, Republic of Korea

^4^Division of Health Policy and Administration, School of Public Health, University of Illinois at Chicago, Chicago, USA

^5^Department of Cardiology, Keimyung University Dongsan Hospital, Keimyung University School of Medicine, Daegu, Republic of Korea

^7^Department of Obstetrics and Gynecology, Keimyung University Dongsan Hospital, Keimyung University School of Medicine, Daegu, Republic of Korea

***Correspondence:**

Chi-Heum Cho, MD, PhD

Director of Covid-19 Task Force Team of Keimyung University Daegu Dongsan Hospital,

Department of Obstetrics and Gynecology, Keimyung University Dongsan Hospital

1035, Dalgubeol-daero, Dalseo-gu, Daegu, Republic of Korea, 42601

Tel: 82-53-258-6000

Fax: 82-53-258-6008

E-mail: [chcho@kmu.ac.kr](mailto:chcho@kmu.ac.kr)

ORCID ID: 0000-0002-0437-4099

Supplementary Table 1. Demographics and characteristics of COVID-19 patients in the development cohort*

|  | **Overall**  **(N=421)** | **Stable**  **(N=384)** | **Progression**  **(N=37)** | **P value** |
| --- | --- | --- | --- | --- |
| Age, years | 56 (44, 68) | 56 (41.5, 66) | 72 (63, 79) | < 0.001 |
| Age group |  |  |  | < 0.001 |
| < 50 years | 142 (33.7%) | 141 (36.7%) | 1 (2.7%) |  |
| 50-59 years | 100 (23.8%) | 94 (24.5%) | 6 (16.2%) |  |
| 60-69 years | 87 (20.7%) | 78 (20.3%) | 9 (24.3%) |  |
| 70-79 years | 68 (16.2%) | 56 (14.6%) | 12 (32.4%) |  |
| ≥ 80 years | 24 (5.7%) | 15(3.9%) | 9(24.3%) |  |
| Gender, male | 117 (27.8%) | 98 (25.5%) | 19 (51.4%) | < 0.001 |
| Comorbidities | 189 (44.9%) | 162 (42.2%) | 27 (73.0%) | 0.003 |
| Initial CXR abnormalities | 179 (42.1%) | 149 (38.4%) | 30 (81.1%) | < 0.001 |
| Laboratory findings |  |  |  |  |
| WBC, /μL | 4840 (3900, 6090) | 4840 (3865, 6070) | 4730 (4320, 6110) | 0.47 |
| ALC, /μL | 1580 (1210, 1900) | 1590 (1240, 1920) | 1250 (930, 1590) | < 0.001 |
| ANC, /μL | 2780 (1980, 3680) | 2690 (1950, 3565) | 3230 (2590, 4240) | 0.003 |
| Hemoglobin, g/L | 12.6 (11.8, 13.7) | 12.6 (11.8, 13.7) | 12.8 (11.8, 13.8) | 0.747 |
| Platelet count, 10^3^/uL | 219 (178, 275) | 220 (178, 276) | 199 (150, 271) | 0.147 |
| BUN, mg/dL | 13 (11, 16) | 13 (11, 16) | 17 (14, 20) | < 0.001 |
| EPI eGFR, mL/min/1.73m^2^ | 96.6 (83.1, 107.3) | 97.4 (84.5, 108.2) | 81.1 (69.1, 90.7) | < 0.001 |
| AST, U/L | 22 (18, 28) | 22 (18, 27) | 25 (19, 38) | 0.008 |
| ALT, U/L | 19 (14, 29) | 19 (14, 28) | 21 (15, 32) | 0.299 |
| Albumin, g/dL | 4.1 (3.8, 4.3) | 4.1 (3.9, 4.4) | 3.8 (3.5, 4.1) | < 0.001 |
| CRP, mg/dL | 0.2 (0.1, 1.1) | 0.2 (0.03, 0.8) | 2.6 (0.6, 7.4) | < 0.001 |
| CPK, U/L | 59 (44, 82) | 59 (44, 81) | 71 (53, 100) | 0.083 |
| LDH, U/L | 430 (372, 510) | 424 (366, 494) | 554 (458, 724) | < 0.001 |
| LDH group |  |  |  | < 0.001 |
| < 500 U/L | 304 (72.2%) | 292 (76.0%) | 12 (32.4%) |  |
| 500-700 U/L | 93 (22.1%) | 79 (20.6%) | 14 (37.8%) |  |
| ≥ 700 U/L | 24 (5.7%) | 13 (3.4%) | 11 (29.7%) |  |

ALC, absolute lymphocyte count; ALT, Alanine aminotransferase; ANC, absolute neutrophil count; AST, Aspartate aminotransferase; BUN, blood urea nitrogen; CPK, Creatinine phosphokinase; CRP, C-reactive protein; CXR, chest x-ray; eGFR, estimated glomerular filtration rate; LDH, lactate dehydrogenase;. WBC, white blood cell.

*Continuous variables were expressed as median (interquartile range) and categorical variables were expressed as number (percentage).

Supplementary Table 2. Demographics and characteristics of COVID-19 patients in the validation cohort*

|  | **Overall**  **(N=140)** | **Stable**  **(N=116)** | **Progression**  **(N=24)** | **P value** |
| --- | --- | --- | --- | --- |
| Age, years | 57 (46, 68) | 56 (44, 66) | 66 (58, 74) | 0.007 |
| Age group |  |  |  | 0.006 |
| < 50 years | 46 (32.9%) | 43 (37.1%) | 3 (12.5%) |  |
| 50-59 years | 36 (25.7%) | 30 (25.9%) | 6 (25.0%) |  |
| 60-69 years | 30 (21.4%) | 22 (19.0%) | 8 (33.3%) |  |
| 70-79 years | 22 (15.7%) | 19 (16.4%) | 3 (12.5%) |  |
| ≥ 80 years | 7 (4.3%) | 2 (1.7%) | 4 (16.7%) |  |
| Gender, male | 35 (25.0%) | 29 (25.0%) | 6 (25.0%) | > 0.999 |
| Comorbidities | 61 (43.6%) | 49 (42.2%) | 12 (50.0%) | 0.485 |
| Initial CXR abnormalities | 61 (43.6%) | 45 (38.8%) | 16 (66.7%) | 0.012 |
| Laboratory findings |  |  |  |  |
| WBC, 10^3^/μL | 5060 (4005, 6155) | 5025 (4005, 6155) | 5640 (4060, 6535) | 0.399 |
| ALC, /μL | 1520 (1260, 1840) | 1585 (1385, 1900) | 1215 (840, 1500) | < 0.001 |
| ANC, /μL | 2855 (2115, 3865) | 2815 (2075, 3780) | 3655 (2490, 4890) | 0.026 |
| Hemoglobin, g/L | 12.7 (11.9, 13.5) | 12.7 (11.9, 13.5) | 12.8 (11.5, 13.8) | 0.927 |
| Platelet count, 10^3^/uL | 233 (173, 280) | 235 (182, 283) | 177 (146, 260) | 0.039 |
| BUN, mg/dL | 13.0 (10.0, 16.0) | 13.0 (10.0, 15.0) | 12.5 (9.5, 21.0) | 0.545 |
| EPI eGFR, mL/min/1.73m^2^ | 95.4 (82.8, 106.6) | 97.5 (86.3, 109.2) | 85.3 (69.2, 100.4) | 0.003 |
| AST, U/L | 22.0 (17.0, 29.5) | 20.0 (17.0, 28.5) | 29.5 (23.0, 43.5) | 0.001 |
| ALT, U/L | 18.0 (14.0, 27.5) | 18.0 (14.0, 27.0) | 23.0 (16.0, 33.0) | 0.269 |
| Albumin, g/dL | 4.1 (3.8, 4.3) | 4.1 (3.9, 4.3) | 3.9 (3.6, 4.2) | 0.020 |
| CRP, mg/dL | 0.30 (0.10, 1.15) | 0.20 (0.03, 0.80) | 1.85 (0.75, 9.30) | < 0.001 |
| CPK, U/L | 58.0 (44.0, 99.5) | 55.5 (43.0, 92.0) | 78.5 (56.5, 131.5) | 0.012 |
| LDH, U/L | 444 (383, 525) | 435 (377, 510) | 538 (437, 863) | < 0.001 |
| LDH group |  |  |  | < 0.001 |
| < 500 U/L | 94 (67.1%) | 84 (72.4%) | 10 (41.7%) |  |
| 500-700 U/L | 33 (23.6%) | 27 (23.3%) | 6 (25.0%) |  |
| ≥ 700 U/L | 13 (9.3%) | 5 (4.3%) | 8 (33.3%) |  |

ALC, absolute lymphocyte count; ALT, Alanine aminotransferase; ANC, absolute neutrophil count; AST, Aspartate aminotransferase; BUN, blood urea nitrogen; CPK, Creatinine phosphokinase; CRP, C-reactive protein; CXR, chest x-ray; eGFR, estimated glomerular filtration rate; LDH, lactate dehydrogenase;. WBC, white blood cell.

*Continuous variables were expressed as median (interquartile range) and categorical variables were expressed as number (percentage).

Supplementary Figure 1. Prediction of severe COVID-19 in categorical risk factors. (A) development cohort and (B) validation cohort


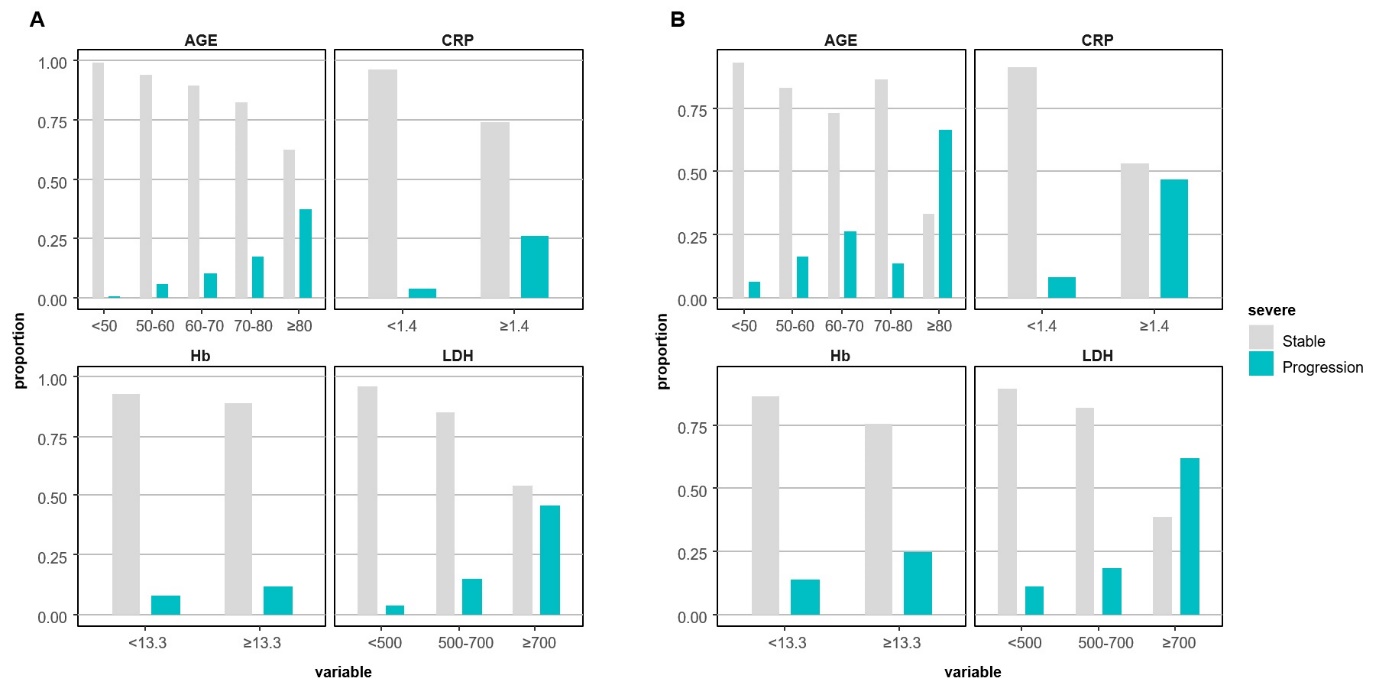

Supplement: Supplementary file 1 — Supplementary Information. [file 41598_2022_7610_MOESM1_ESM.docx]
